# Panaxadiol Saponin and Dexamethasone Improve Renal Function in Lipopolysaccharide-Induced Mouse Model of Acute Kidney Injury

**DOI:** 10.1371/journal.pone.0134653

**Published:** 2015-07-31

**Authors:** Yan Chen, Yanwei Du, Yang Li, Xiaoqin Wang, Pin Gao, Guang Yang, Yuan Fang, Yan Meng, Xuejian Zhao

**Affiliations:** 1 Department of Pathophysiology, College of Basic Medicine, Jilin University, Changchun, China; 2 Department of Nephropathy, The First Hospital of Jilin University, Changchun, China; University of Kentucky, UNITED STATES

## Abstract

**Background:**

Acute kidney injury (AKI) is a serious complication of systemic inflammatory response syndrome (SIRS), which has a high mortality rate. Previous studies showed that panaxadiol saponin (PDS) and Dexamethasone have similar anti-inflammatory properties and protect cardiopulmonary function in lipopolysaccharide (LPS)-induced septic shock rats. In the present study, we investigated whether PDS or Dexamethasone has a similar role in improving kidney function in LPS-induced AKI mice.

**Methods and Results:**

Mice subjected to LPS (10 mg/kg) treatment exhibited AKI demonstrated by markedly increased blood urea nitrogen and creatinine levels compared with controls (P<0.01). However, PDS and Dexamethasone induce similar reverse effects on renal function, such as reduced serum creatinine and blood urea nitrogen levels compared with the LPS group (P<0.05). PDS decreased the production and release of tumor necrosis factor (TNF)-α and interleukin (IL)-6 by inhibiting the NF-κB signaling pathway, down-regulating inducible nitric oxide synthase protein expression levels and inhibiting oxidative stress. In most anti-AKI mechanisms, PDS and dexamethasone were similar, but PDS are better at inhibition of TNF production, promote SOD activity and inhibition of IKB phosphorylation. In addition, nuclear glucocorticoid receptor expression was markedly enhanced in PDS and Dexamethasone treatment groups. Further research is required to determine whether PDS can combine with the glucocorticoid receptor to enter the nucleus.

**Conclusion:**

This study demonstrated that PDS and dexamethasone have similar reverse amelioration for renal functions, and have potential application prospects in the treatment of sepsis-induced AKI.

## Introduction

Renal failure during gram negative sepsis can be profound, difficult to treat and fatal [1]. Acute kidney injury (AKI) occurs during endotoxemia, where endotoxin binds to endothelium and leukocytes, inducing the production and release of cytokines and a systemic “cytokine storm”, namely systemic inflammatory response syndrome (SIRS). This is accompanied by decreased peripheral vascular resistance and hypotension leading to septic shock [1, 2, 3]. During this systemic disturbance, a puzzling aspect of AKI in sepsis is the paucity of structural renal damage despite severely impaired function [2, 3]. Studies have shown that renal damage in experimental septic AKI is potentially reversible, at least as inferred from the benefits afforded by early interventions that restore renal function. Such interventions include volume replacement, free radical scavengers and anti-inflammatory therapies [2, 4]. Wang et al. demonstrated that antioxidant therapy could reverse renal glomerular filtration rate (GFR) and renal blood flow (RBF) during endotoxemic acute renal failure (ARF) [5]. Gupta et al. found that activated protein C (APC) exhibited anti-inflammatory properties, modulated endothelial functions, down-regulated renal inducible nitric oxide synthase (iNOS) and modulated the renin-angiotensin system, resulting in APC improving renal function in LPS-induced AKI rats [6]. Hsing et al. demonstrated that Propofol treatment protected kidneys from sepsis-induced AKI by decreasing inflammatory cytokines and inhibiting oxidative stress [7]. Thus, early anti-inflammatory and antioxidant therapy can improve renal function.

Based on these previous studies, AKI is a serious complication of SIRS. Both TNF-α and LPS have direct pro-inflammatory effects on tubules [1, 8], and LPS directly induces TNF-α expression in tubules [9]. LPS and TNF-α can bind directly to endothelium, leukocytes and other cell types to produce and release cytokines that induce SIRS accompanied by activation of TLR4-mediated nuclear factor NF-κB signaling pathways [10, 11]. In addition, LPS directly induces TNF-α expression that synergizes with other stressors to promote the tubular production of toxic cytokines [12]. Thus, primary tubule lesions might be induced by LPS/TNF-α [1].

Molecular mechanisms of renal microvascular and tubular injury and the role of reactive nitrogen-oxygen have been suggested by *in vivo* and *in vitro* studies where exposure of animals or renal cells to LPS induced inflammatory responses and free radicals, including reactive oxygen species (ROS) and nitric oxide (NO) [7]. Antioxidants can protect against AKI caused by oxidative stress in murine models of endotoxemia [5, 6].Wu et al. showed that selective iNOS inhibition by L-N6-(1-Iminoethyl) lysine (L-NIL) abolished tubule oxidant stress and corrected microcirculatory abnormalities [13].

Ginseng radix has been used since ancient times to increase vitality for resuscitation. Panaxadiol Saponins (PDS) is an extract of ginseng stem and leaves, has anti-shock and organ protective effects when an organism is in stress. Our previous studies found that PDS and Glucocorticoids (GCs) have similar anti-shock effects in rat and dog models[14, 15]. Recently, Choi et al. demonstrated that GCs were clinically recommended for the treatment of septic shock, and had favorable effects on septic AKI in several animal experiments [16]. Dixon et al. showed that GCs are one of the most frequently prescribed therapies in rheumatology because of their powerful anti-inflammatory cytokine effect. However, GCs are associated with a wide range of adverse events, particularly at higher doses [17], including stress hemorrhages and osteoporosis. A recent case-control study observed that oral GC use was associated with an increased risk of acute pancreatitis [18]. Our previous studies found that PDS and Dexamethasone had similar effects in down-regulating the expression of IκB and inhibiting the expression of NF-κB-p50 and p65 in a rat model of acute lung injury induced by LPS [14].

In the present study, we show that PDS and Dexamethasone similarly decreased the production and release of pro-inflammatory cytokines (TNF-α and IL-6) by inhibiting NF-κB signaling pathways, suppressed the formation of reactive nitrogen-oxygen species, and up-regulated the activity of superoxide dismutase (SOD). In addition, PDS promoted the trafficking of the glucocorticoid receptor into the nucleus. Therefore, our data suggest that PDS and Dexamethasone had similar beneficial effects on renal function in LPS-induced AKI mice.

## Materials and Methods

### Animals

The experimental protocol was approved by the Animal Ethics Review Committee of Jilin University. Animal experiments were performed in accordance with the regulations set by the Institutional Committee for the Care and Use of Laboratory Animals of the Experimental Animal Center of Jilin University, China. C57BL/6 mice were purchased from the animal center of Jilin University and housed on a 12-h:12-h light/dark cycle and were kept in air-conditioned rooms at 22 ± 2°C, unless stated otherwise and were allowed free access to food and water.

### AKI mouse model

Thirty-two male C57BL/6 mice (weight 20 ± 4g) were randomly divided into four groups (n = 8 per group) as follows: control group, LPS group, PDS plus LPS group, and Dexamethasone plus LPS group. LPS was phenol extracted from Escherichia coli serotype O111:B4 (Sigma, Poole, UK). PDS is a patent product (ZL 98100070.3), provided from Laboratory of Natural Medicine Research, Jilin University, China. Dexamethasone was provided by SanDong-XinHua Pharmaceuticals. Co. Ltd. The control group was injected with 0.5 ml 0.85% NaCl intraperitoneally. LPS group, PDS plus LPS group, and Dexamethasone plus LPS groups mice were injected intraperitoneally with LPS (10 mg/kg). In PDS plus LPS and Dexamethasone plus LPS groups, mice were injected intraperitoneally with PDS (25.0 mg/kg) or Dexamethasone (2.5mg/kg) at 1 h before LPS injection. Mice were sacrificed under deep anesthesia with pentobarbital 12 h after LPS injection. Blood was withdrawn from the heart and kidneys were collected for biochemical and molecular assays. Because animals injected with LPS underwent oliguria, urine analysis was not performed.

### Blood biochemical analysis

Serum samples from mice were prepared by centrifugation (3,000 rpm for 10 min) and analyzed for the following blood biochemistry parameters: kidney markers [creatinine, blood urea nitrogen (BUN)], hepatic markers [aspartate aminotransferase (AST), and alanine aminotransferase (ALT)]. Diagnostic kits (Nanjing Jiancheng Bioengineering Institute, Nanjing, China) with an automatic biochemistry analyzer (Beckman Coulter LX20, Beckman, USA) were used.

### Analysis of serum inflammatory cytokines

The concentration of TNF-α and IL-6 in serum was quantified using an enzyme linked immunoassay kit (Nanjing Jiancheng Bioengineering Institute, Nanjing, China). The results were expressed as pg per gram of wet tissue (pg/mg tissue).

### Detection of Malondialdehyde (MDA) levels in kidney tissue

Kidney tissue homogenates (0.01 M PBS buffer to produce a 10% tissue lysate) were used to measure kidney MDA levels by the thiobarbituric acid (TBA) method. The MDA-TBA adduct produced during the reaction of MDA in samples with TBA was detected spectrophotometrically at 535 nm (UV-2401PC, Shimadzu, Japan). The result was expressed as nmol per milligram of protein (nmol/mg.prot). MDA detection kits were provided by Nanjing Jiancheng Bioengineering Institute (Nanjing, China).

### Determination of superoxide dismutase (SOD) enzyme activity

Kidney superoxide dismutase (SOD) activity was determined using a SOD assay kit (Nanjing Jiancheng Bioengineering Institute, Nanjing, China) in accordance with the manufacturer’s instructions. The result was expressed as units per milligram of protein (U/mg prot).

### Detection of NO content in kidney tissue

NO content in kidney tissue was assayed using the Nitrate reductase enzymatic method according to the manufacturer’s instructions (Nanjing Jiancheng Bioengineering Institute, Nanjing, China). Distilled water was used for the zero reading, then the content of NO in serum samples was detected at 550 nm by spectrophotometer and the NO content expressed as μmol/g protein.

### Western blot analysis

Cytoplasmic and nuclear proteins were extracted with an NEPER Nuclear and Cytoplasmic Extract kit (Pierce, Rockford, IL, USA) according to the manufacturer’s instructions. Briefly, 100 mg of kidney was homogenized in CER I Reagent using a glass Teflon homogenizer. Then CER II Reagent was added to the homogenates. After incubation on ice for 1 min, the homogenates were centrifuged at 12,000 ×*g* for 5 min at 4°C. The resulting supernatant was collected (cytoplasmic extraction) and the pellet was resuspended in ice-cold (NER). The nuclear suspension was placed on ice with continued vortexing for 15 s every 10 min for a total of 40 min, and then centrifuged at 12,000 ×*g* for 10 min at 4°C. The supernatant (nuclear extraction) was collected and stored at −70°C until use.

Total protein concentration in cytoplasmic and nuclear extractions was determined by the bicinchoninic acid (BCA) method (Pierce). A total of 30 μg protein mixed with SDS-PAGE buffer was loaded on 10% sodium dodecyl sulfate-polyacrylamide gels for electrophoresis as described previously [14]. Separated proteins were then transferred to PVDF membranes (Millipore, Bedford, MA, USA). Blots were incubated with polyclonal antibodies against mitochondrial superoxide dismutase-2 (Mn-SOD), iNOS, Glucocorticoid Receptor(GR), p-IκB, IκB, p50, p65, Lamin B1 (Abcam, Cambridge, MA, USA) and β-actin (Proteintech Group, WuHan, China). Horseradish peroxidase (HRP)-labeled anti-rabbit IgG secondary antibodies (Proteintech Group, SA00001-2, dilution 1:10,000) and blots were prepared using Pierce ECL Plus Kit (Thermo Fisher Scientific Inc., Rockford, IL, USA) and then all blots were exposed to Thermo Scientific CL-XPosure Film* (Part No. 34090). Relative protein expression levels were quantified by optical density analysis and normalized to β-actin or Lamin B1 (only for nuclear proteins).

### Statistical analysis

All quantitative values are expressed as mean ± SEM. Statistical differences between two groups were examined by Student’s *t-*test using SPSS version 17.0. P-values less than 0.05 were considered statistically significant.

## Results

### Dexamethasone-like protective effects of PDS treatment on kidney index and kidney functional markers in LPS-injected mice

**Fig 1:**
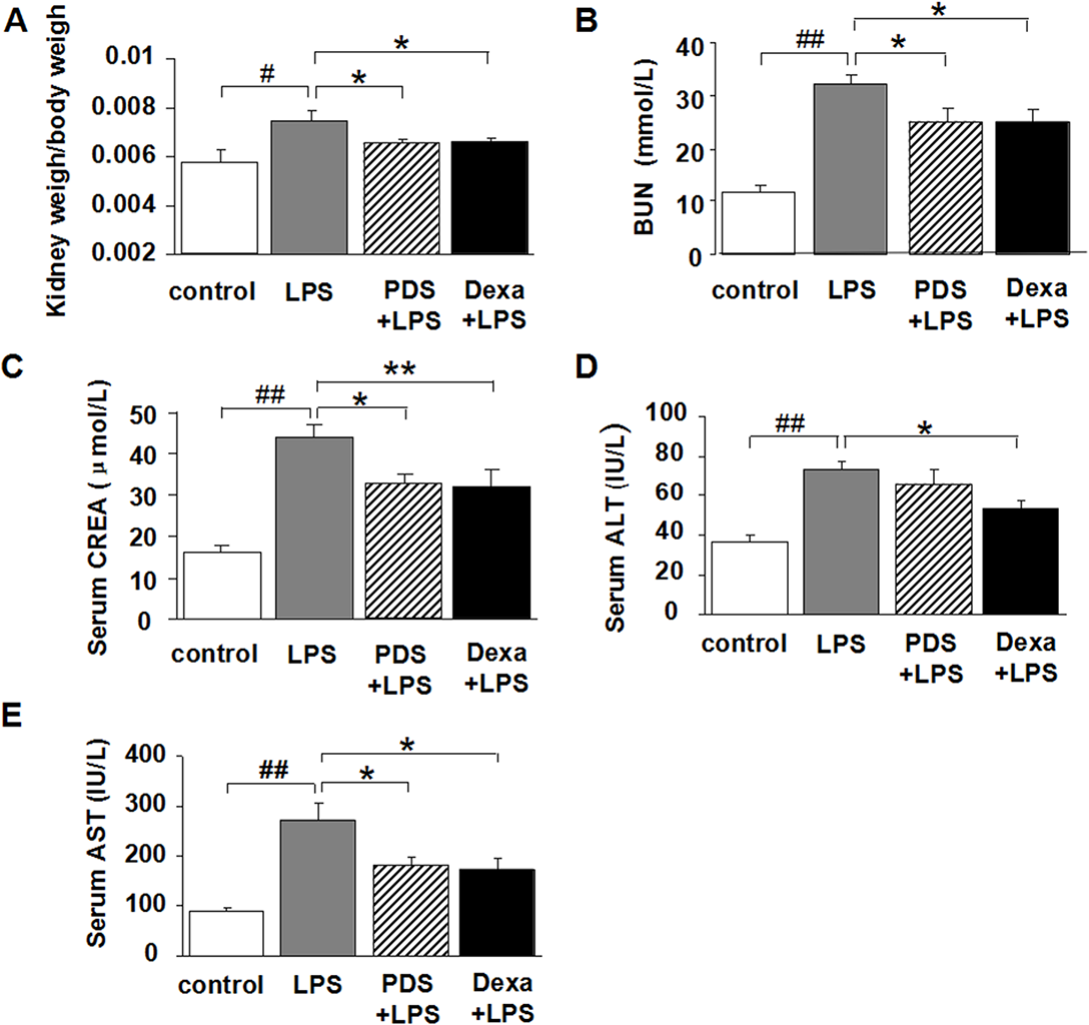
Effects of PDS on kidney, hepatic and cardiac markers in mice. A: Kidney index = kidney weight/body weight; B:Serum Blood Urea Nitrogen (BUN); C: Serum creatinine (CREA); D: Serum alanine transaminase (ALT); E: Serum aspartate aminotransferase (AST). LPS: lipopolysaccharide; PDS: Panaxadiol saponins; Dexa: dexamethasone. Data represent mean ± SEM. ^#^P<0.05 and ^##^P<0.01 vs. control group; *P<0.05 and **P<0.01 vs. LPS group (n = 8 per group).

The kidney index of mice, the level of serum urea (BUN) and creatinine(CREA) were shown in Fig 1A, 1B and 1C. LPS treatment induced a significant increase in the relative weight of kidneys compared with the control group (0.0073 ± 0.0004 vs 0.0056 ± 0.0005; P<0.01), whereas treatment with PDS and Dexamethasone to LPS-injected mice diminished this increase (0.0064 ± 0.0001 and 0.0065 ± 0.0001; P<0.05). As shown in Fig 1B and 1C, serum levels of creatinine (44.15 ± 2.87 vs 16.16 ± 1.43) and BUN (32.31 ± 1.76 vs 11.83 ± 0.99; P<0.01) were significantly higher in the LPS-treated animals compared with the control group. Co-treatment of the animals with PDS (25 mg/kg) significantly reduced the high level of serum creatinine (32.60 ± 2.80; P<0.05) and BUN (24.95 ± 2.61; P<0.05), similar to the effect of Dexamethasone co-treatment (CREA 31.77 ± 4.60; BUN 24.90 ± 2.41).

### Protective effects of PDS treatment on hepatic functional markers in LPS-injected mice

The activities of AST and ALT were determined in serum samples as liver function markers (Fig 1D and 1E). In the control group, LPS-induced liver injury was measured by significantly increased liver ALT (73.29 ± 4.00 vs 36.37 ± 3.61; P<0.01) and AST levels (273.14 ± 32.85 vs 90.50 ± 7.53; P<0.01). This result suggested that liver function markers are elevated in the serum because of the release of the enzymes from damaged liver. Conversely, PDS and Dexamethasone treatment with LPS injections caused significantly decreased AST levels (181.00 ± 16.54 and 173.42 ± 20.63, respectively) compared with LPS alone (P<0.05). Dexamethasone also caused a similar reduction in ALT levels.

### PDS lowers TNF-α and IL-6 levels in serum of LPS-injected mice

**Fig 2:**
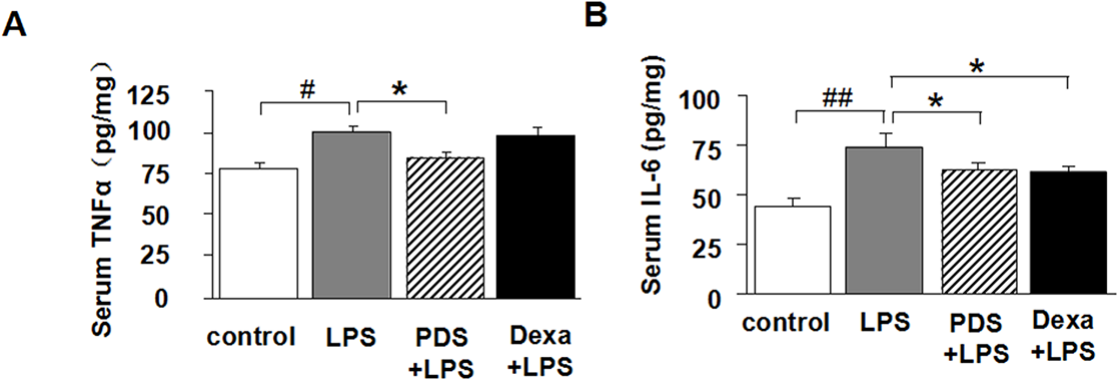
Serum levels of inflammatory cytokines. TNF-α (A) and IL-6 (B) in serum from mice treated with PDS or Dexamethasone after LPS injection (n = 8 per group). LPS: lipopolysaccharide; PDS: Panaxadiol saponins; Dexa: dexamethasone. Data represent mean ± SEM. ^#^P<0.05 and ^##^P<0.01 vs. control group; *P<0.05 vs. LPS group (n = 8 per group).

TNF-α and IL-6 levels in the LPS-treated group were significantly higher than in the control group (P<0.05 and P<0.01, Fig 2). The anti-inflammatory effect of PDS was accompanied by a decrease in TNF-α and IL-6 levels in the PDS+LPS groups compared with the LPS group (P<0.05). Dexamethasone significantly reduced IL-6 levels (P<0.05).

### Suppressive effects of PDS on activation of the NF-κB pathway

**Fig 3:**
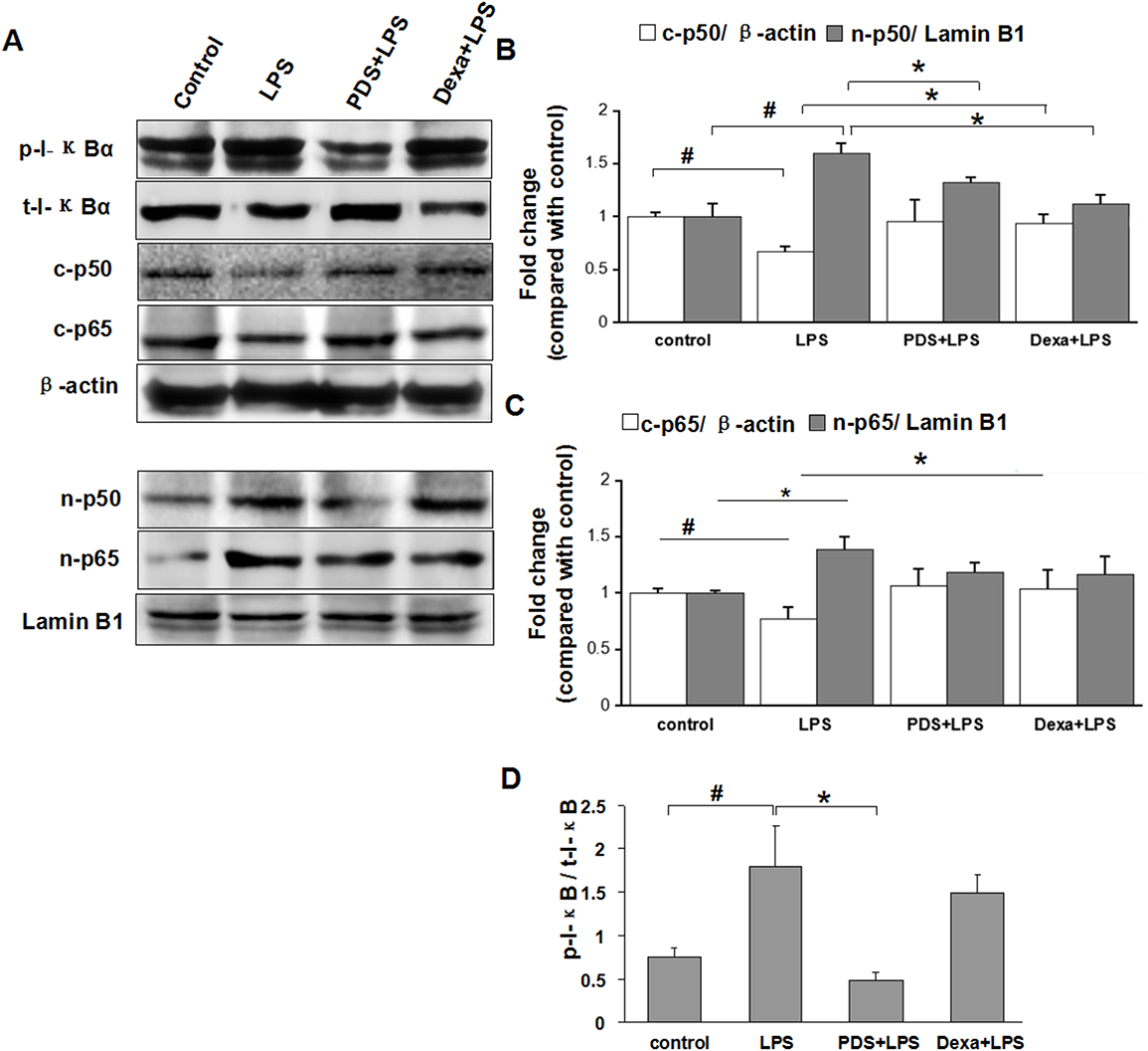
Western blot analysis of IκBα and NF-κB expression and activation in kidney. A: Expression of phosphorylated (t-p-IκBα) and total IκBα (t-IκBα), cytosolic (c-p50, c-p65) and nuclear p50 and p65 (n-p50, n-p65) in kidney tissue detected by western blotting. B: Density ratio of c-p50 with β-actin and n-p50 with Lamin B1. C: Density ratio of c-p65 with β-actin and n-p65 with Lamin B1 D: Density ratio of p-IκB and t-IκB. IκBα: inhibitor κBα; NF-κB: nuclear factor kappa B, LPS: lipopolysaccharide; PDS: Panaxadiol saponins; Dexa: dexamethasone. Data represent mean ± SEM. ^#^P<0.05 vs. control group; *P<0.05 vs. LPS group (n = 3 per group).

The transcription factor NF-κB plays a key role in inflammatory diseases. We investigated the nuclear translocation of p50 and p65 as surrogate markers for NF-κB pathway activation. Analysis of the control group demonstrated that NF-κB p65 and p50 were predominantly localized in the cytoplasm (Fig 3A, 3B and 3C). When the NF-κB pathway was activated in the LPS group, translocation of NF-κB p65 and p50 into the nucleus was observed. In PDS+LPS and Dexamethasone+LPS groups, levels of NF-κB p50 in the nucleus were reduced when compared with the LPS group (Fig 3B and 3C; P<0.05). Although Dexamethasone and PDS had a tendency to decrease nuclear translocation of p65 compared with the LPS group, this was not significant (P>0.05).

Inhibitor I-κB binds with NF-κB to inhibit its activation. Phosphorylation of I-κB releases NF-κB allowing it to translocate into the nucleus. When mice were treated with LPS, about 2/3 I-κB were phosphorylated (P<0.05) (Fig 3A and 3D). Co-treatment with PDS markedly reduced the ratio of phosphorylated I-κB/t-I-κB (P<0.05), and PDS increased the expression of t-I-κB (P<0.05).

### PDS reduced NO levels by inhibition of iNOS expression in kidney tissues of LPS-injected mice

**Fig 4:**
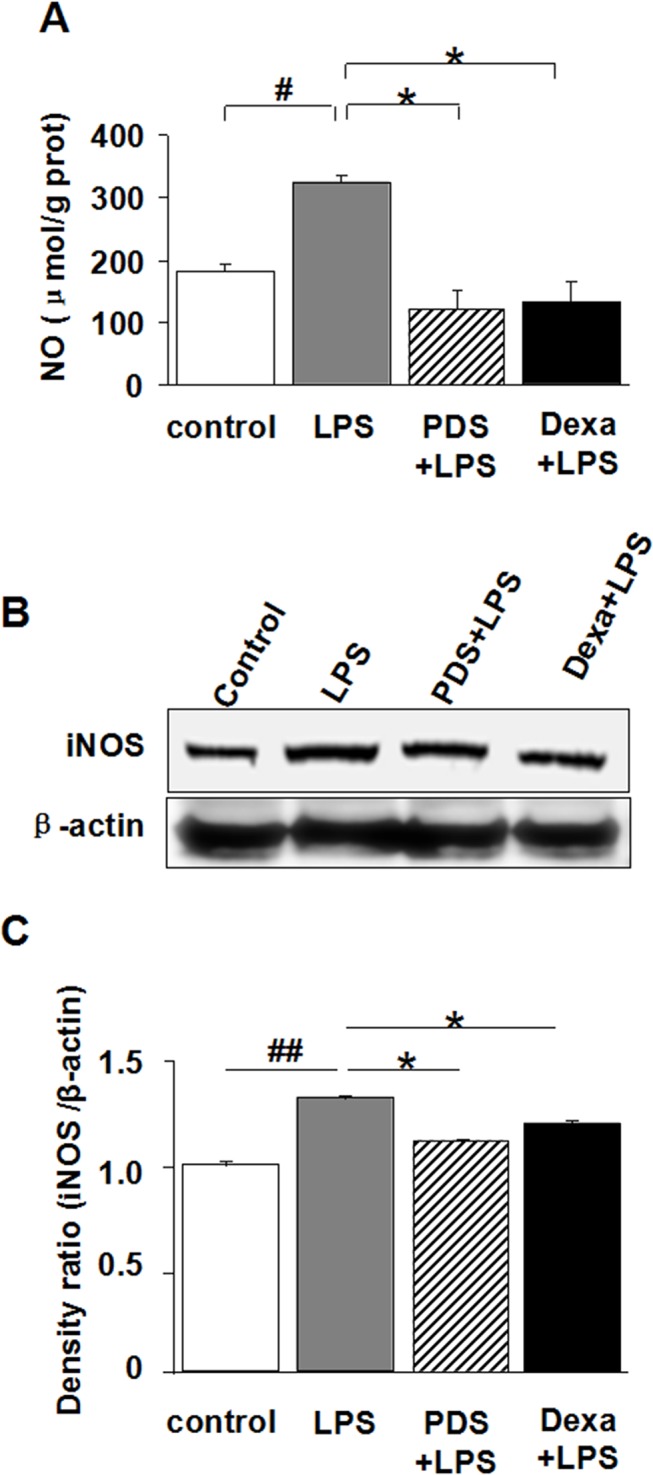
Antioxidant effect of PDS on LPS-induced NO in kidney. A: Inhibitory effect of PDS on NO synthesis in kidney tissues after LPS injection. B: Expression of iNOS in kidney tissue detected by western blotting. C: Density ratio of iNOS with β-actin. LPS: lipopolysaccharide; PDS: Panaxadiol saponins; Dexa: dexamethasone. Data represent mean ± SEM. ^#^P<0.05 and ^##^P<0.01 vs. control group; *P<0.05 vs. LPS group (n = 8 for A, or n = 3 for B,C, per group).

At 12 h after injection, LPS-induced a sharp increase of NO level in kidney tissues, as a result of up-regulated iNOS expression (Fig 4A, 4B and 4C; P<0.05). This inductive NO synthesis was reversed by PDS or Dexamethasone treatment (P<0.05), as a concomitant result of reduced iNOS expression (Fig 4; P<0.05).

### PDS reduced MDA levels and increased SOD activity in kidney tissues of LPS-injected mice

**Fig 5:**
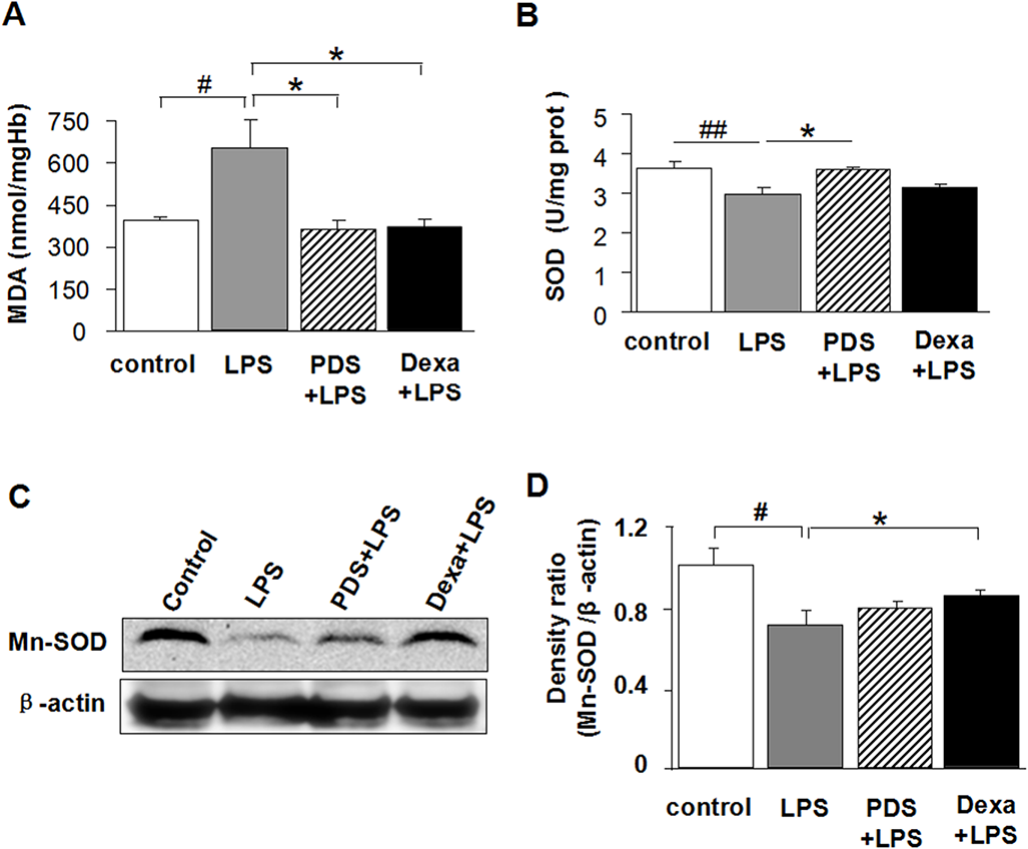
Antioxidant effect of PDS on LPS-induced oxidative stress in kidney. A: The levels of MDA, B: The activities of SOD (C) in kidney tissues of mice. C: Expression of Mn-SOD in kidney tissues detected by western blotting. D: Density ratio of Mn-SOD with β-actin. LPS: lipopolysaccharide; PDS: Panaxadiol saponins; Dexa: dexamethasone. Data represent mean ± SEM. ^#^P<0.05 and ^##^P<0.01 vs. control group; *P<0.05 vs. LPS group (n = 8 for A,B, or n = 3 for C,D, per group).

At 12 h after mice were injected with LPS, MDA levels in kidney tissues were significantly higher compared with control (P< 0.05; Fig 5A). The antioxidant effect of PDS was accompanied by a decrease in MDA levels in the PDS+LPS group compared with the LPS group (P<0.05), similar to changes in the Dexamethasone group. The ROS clearance ability was determined by SOD activity in kidney tissues (Fig 5B). LPS inhibited SOD activity at 12 h after injection (P<0.01). PDS treatment significantly recovered SOD activity (P<0.05 vs LPS group). Dexamethasone treatment also had such trend, even no significant. The expression levels of Mn-SOD in kidney tissues also reduced markedly by treatment of LPS. Furthermore, LPS injection reduced Mn-SOD expression levels that were reversed by Dexamethasone treatment (Fig 5C and 5D; P<0.05). PDS had a similar tendency.

### PDS promoted GR protein translocation from the cytoplasm to the nucleus in kidney tissues of LPS-injected mice

**Fig 6:**
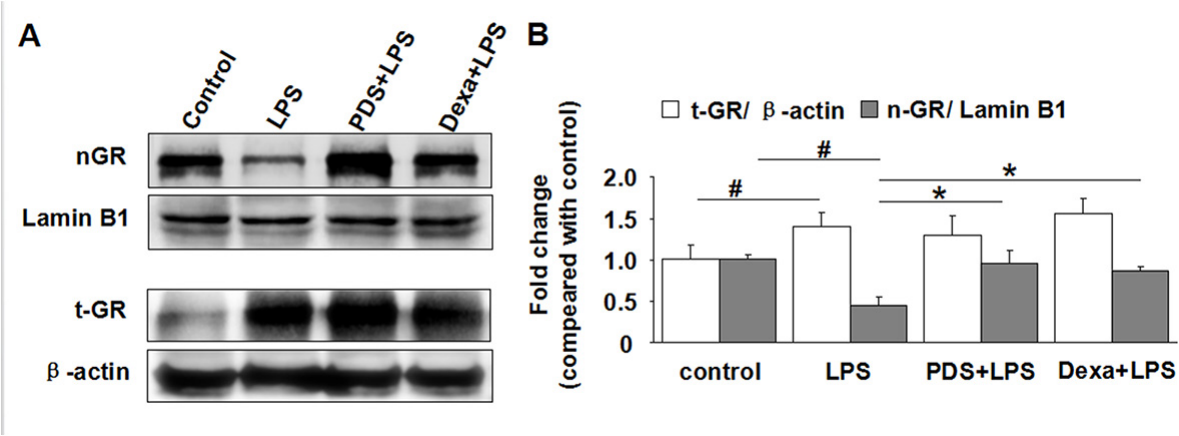
Western blot analysis of expression and nuclear translocation of GR in kidney. A: Expression of nuclear (nGR) and total GR (tGR) in kidney tissues detected by western blotting. B: Density ratio of nGR with β-actin and tGR with Lamin B1. GR: glucocorticoid receptor; LPS: lipopolysaccharide; PDS: Panaxadiol saponins; Dexa: dexamethasone. Data represent mean ± SEM. ^#^P<0.05 vs. control group; *P<0.05 vs. LPS group (n = 3 per group).

A single prominent band was detected at approximately 95 kD, in total kidney and nuclear proteins. Fig 4A indicates the change in GR protein expression in kidney tissues after LPS treatment with PDS or Dexamethasone. Total GR expression increased in animals treated with LPS compared with control animals (Fig 6A and 6B; P<0.05). Although total GR protein increased in the LPS group, nuclear protein was decreased (Fig 6A and 6B), indicating that less GR translocation from the cytoplasma to the nucleus had occurred. Co-treatment of PDS or Dexamethasone with LPS showed no significant change in total GR protein levels. However, a marked increase of nuclear GR protein in mice treated with PDS or Dexamethasone was observed compared with LPS group mice (Fig 6A and 6B; P<0.05).

## Discussion

Septic AKI is a serious complication of SIRS. LPS and TNF-α directly bind to endothelium, leukocytes and other cell types to produce cytokines and induce SIRS accompanied by activation of Toll-like receptor IV-mediated nuclear factor NF-κB signaling [10, 11]. Studies demonstrated that renal damage in experimental septic AKI is potentially reversible [5–7]. This study demonstrated that 12 h after LPS (10 mg/kg) administration, serum creatinine level and BUN contents were significantly elevated compared with controls (P<0.01). However, PDS and Dexamethasone treatment groups significantly reduced serum creatinine and BUN levels compared with the LPS group (P<0.05). The pathogenesis of AKI secondary to endotoxemia, is thought to derive from pro-inflammatory responses including cytokine secretion and reactive nitrogen-oxygen species (RNOS) [1, 12, 13]. In this study, serum TNF-α and IL-6 levels significantly increased in the LPS treatment group compared with controls (P<0.05). In contrast, PDS treatment groups showed somewhat anti-inflammatory properties in LPS-induced AKI mice, such as decreased serum TNF-α and IL-6 levels compared with the LPS group. TNF-α is a potent pro-inflammatory cytokine exerting pleiotropic effects on various cell types and has a critical role in the pathogenesis of inflammatory diseases [12]. This study demonstrated that PDS down-regulated the expression of p-IκB and inhibited the expression of NF-κB-p50 and p65 in LPS-induced AKI. We speculate that PDS suppressed TNF-α and IL-6 production by the NF-KB signaling pathway.

Holthoff et al. showed that resveratrol protected against septic AKI by decreasing the activity of reactive nitrogen species (RNS) [19]. Wu et al. showed that selective iNOS inhibition by L-N6-(1-Iminoethyl) lysine (L-NIL) abolished tubule oxidant stress and corrected microcirculatory abnormalities [20]. In the current study, we found that PDS and Dexamethasone similarly down-regulated the expression of iNOS in nephridial tissues compared with the LPS group (P<0.05), and that they had abilities to increase serum SOD activity or increase SOD expression, thenreduce serum MDA content. Thus, PDS and Dexamethasone have similar anti-oxidative stress properties in LPS-induced AKI. Studies have shown that LPS directly stimulates ROS generation in mesangial cells, which can damage tubular cells during sepsis [7]. Kruzel et al. showed that LPS-induced oxidative burst was of mitochondrial origin and that the release of ROS was localized to complex III in the respiratory chain [21]. The current study showed that mitochondrial-specific Mn-SOD expression levels in the LPS group were significantly decreased compared with controls (P<0.01). However, the PDS and Dexamethasone treatment groups showed the reverse tendency compared with the LPS group.

The close association between capillary dysfunction, RNS generation, and tubular damage suggests that the peritubular capillary/tubular microenvironment plays a critical role in sepsis-induced AKI [22, 8]. Furthermore, studies using the iNOS inhibitor L-NIL suggest that pharmacologic inhibition of iNOS and/or RNS should be considered as a potential therapeutic approach for the prevention of sepsis-induced AKI [19, 23]. Thus, PDS has similar abilities to nitric oxide synthase inhibitors, L-NIL. Therefore, the down-regulation of iNOS-derived NO by PDS and Dexamethasone also may account for improved peritubular capillary flow and renal function, such as significantly lower serum CREA and BUN compared with the LPS group.

Dixon et al. demonstrated GCs are powerful anti-inflammatory cytokine drugs that have associated adverse events [14]. Our previous studies found that PDS and Dexamethasone have similar ameliorating effects on cardiopulmonary functions in animal models of hemorrhagic and endotoxic shock [16]. When the hemorrhagic shock dog model was treated with Dexamethasone, two of 14 dogs died because of stress gastrointestinal hemorrhage. However, PDS treated animals did not die [15]. Thus, PDS and Dexamethasone have a similar ability to restore renal function through anti-inflammatory effects and resistance to oxidative stress in LPS-induced AKI mice. Interestingly, PDS are better at inhibition of TNF production, promote SOD activity and inhibition of IKB phosphorylation.

In addition, we observed that glucocorticoid nucleus receptor expression was enhanced in PDS and Dexamethasone treatment groups. Lee et al. found that Ginsenoside Rg1 is a functional ligand of GR [24]. PDS might have a similar role to Rg1, but this requires further study.
